# Prognostic Impact of Ectopic Fat Deposition Within the Psoas Muscle in Patients with Stage IV Pancreatic Cancer Receiving Systemic Chemotherapy

**DOI:** 10.3390/jcm15103936

**Published:** 2026-05-20

**Authors:** İbrahim Çil, İlker Nihat Ökten, Zeynep Nihal Kazcı, Ayberk Bayramgil, Tuba Baydaş, Gözde Balkaya Aykut, Pembegül Yumuştutan, Aziz Batu, Yunus Emre Altintas, Sevde Nur Emir, Fatma Kulalı, Melike Özçelik

**Affiliations:** 1 Department of Medical Oncology, Umraniye Training and Research Hospital, Istanbul 34764, Türkiye; ayberkbayramgil@gmail.com (A.B.); gozdebalkayaa@gmail.com (G.B.A.); yumustutan@gmail.com (P.Y.); azizbatu84@gmail.com (A.B.); yunusaltintas1688@gmail.com (Y.E.A.); drmelike.ozcelik@gmail.com (M.Ö.); 2 Department of Medical Oncology, Göztepe Şehir Hospital, Istanbul Medeniyet University, Istanbul 34722, Türkiye; ilkernihat@gmail.com (İ.N.Ö.); tuba.baydas@gmail.com (T.B.); 3 Department of Radiology, Göztepe Şehir Hospital, Istanbul Medeniyet University, Istanbul 34722, Türkiye; zeynepkazci17@gmail.com; 4 Department of Radiology, Umraniye Training and Research Hospital, Istanbul 34764, Türkiye; drsevdenuremir@gmail.com (S.N.E.); ftkulali@gmail.com (F.K.)

**Keywords:** pancreatic cancer, prognostic biomarker, body composition, myosteatosis, psoas muscle

## Abstract

**Background:** Computed tomography-based body composition parameters are emerging prognostic markers in pancreatic cancer. While sarcopenia and myosteatosis have been widely studied, the prognostic significance of ectopic fat deposition within the psoas muscle remains unclear. We aimed to evaluate the prognostic impact of the fat ratio within the psoas muscle (FRPM) in patients with stage IV pancreatic cancer receiving first-line systemic chemotherapy. **Methods:** This retrospective cohort study included 99 patients with stage IV pancreatic cancer treated with first-line chemotherapy. Baseline CT images at the L3 level were analyzed, and FRPM was calculated as the proportion of intramuscular fat to total psoas muscle area. FRPM was analyzed primarily as a continuous variable. Exploratory low- and high-FRPM groups were defined using sex-specific medians for descriptive comparisons and Kaplan–Meier analyses. Overall survival (OS) and progression-free survival (PFS) were assessed using Kaplan–Meier and Cox regression analyses. Multivariable models were adjusted for age, sex, ECOG performance status, liver metastasis, and C-reactive protein (CRP). **Results:** Among 99 patients, 48 were categorized as having low FRPM and 51 as having high FRPM based on exploratory sex-specific median-based groups. Higher FRPM correlated with older age and higher BMI and inversely correlated with psoas muscle size and PMI. Median OS was 9.76 months in the low-FRPM group versus 5.78 months in the high-FRPM group, and median PFS was 5.29 versus 3.68 months. In the main multivariable Cox model, higher FRPM was associated with worse OS when analyzed as a continuous variable and reported per 1-standard deviation increase (adjusted HR: 1.43, 95% CI: 1.08–1.91, *p* = 0.014). After additional adjustment for first-line treatment-regimen category, the association remained directionally consistent but did not retain conventional statistical significance (adjusted HR: 1.32, 95% CI: 0.99–1.77, *p* = 0.060). **Conclusions:** Higher FRPM was associated with shorter OS and PFS in patients with stage IV pancreatic cancer receiving first-line systemic chemotherapy. These findings suggest that ectopic fat deposition within the psoas muscle may represent a potential CT-based muscle-quality marker associated with adverse prognosis. External validation and comparison with conventional adiposity parameters are required before clinical application.

## 1. Introduction

Pancreatic cancer remains one of the most lethal malignancies, and patients with metastatic disease continue to have limited survival despite improvements in systemic treatment [1]. In this setting, there is a strong need for clinically accessible biomarkers that can better capture host vulnerability and help refine prognostic stratification beyond conventional clinicopathologic variables. Computed tomography (CT)-based body composition analysis has emerged as an attractive approach because it can be derived from routine imaging without additional cost or patient burden [2]. The third lumbar vertebral level (L3) is widely used for this purpose and has become a standard landmark for body composition assessment in oncology [3].

Among body composition parameters, sarcopenia and myosteatosis have attracted growing attention in pancreatic cancer. Recent meta-analyses suggest that CT-defined sarcopenia is associated with inferior survival in pancreatic cancer, although reported effect sizes vary depending on disease stage, treatment setting (curative vs. palliative), and the criteria used to define low muscle mass [4,5,6]. At the same time, a 2024 systematic review and meta-analysis focused specifically on myosteatosis reported that fatty degeneration of skeletal muscle is associated with worse overall and progression-free survival in pancreatic cancer, supporting the idea that muscle quality may carry distinct prognostic information beyond muscle quantity alone [7].

This distinction may be particularly relevant in advanced pancreatic cancer, where cancer cachexia, metabolic dysregulation, reduced physical reserve, and treatment intolerance often coexist [8]. Cancer cachexia is defined by international consensus as a multifactorial syndrome characterized by ongoing skeletal muscle loss, with or without fat loss, that cannot be fully reversed by conventional nutritional support and contributes to progressive functional impairment [9]. In a study of metastatic pancreatic adenocarcinoma treated with first-line chemotherapy, skeletal muscle density and sarcopenia were both associated with prognosis, suggesting that reduced muscle quality may reflect an especially vulnerable host phenotype [10]. More broadly, other pancreatic cancer cohorts have also reported that myosteatosis or related body composition parameters are linked to survival, although the optimal metric has not been established [11,12,13].

Most prior studies have focused on whole-muscle density or global skeletal muscle measurements such as skeletal muscle index. By contrast, the prognostic significance of ectopic fat deposition specifically within the psoas muscle has been much less studied. A 2022 study in stage IV gastric cancer patients receiving systemic chemotherapy reported that the fat ratio within the psoas muscle was associated with prognosis, raising the possibility that psoas-centered intramuscular fat measurements may capture clinically meaningful changes in muscle quality in advanced gastrointestinal malignancies [14]. However, to our knowledge, this specific approach has not been adequately evaluated in stage IV pancreatic cancer treated with systemic therapy.

Accordingly, the aim of the present study was to investigate the prognostic impact of the fat ratio within the psoas muscle (FRPM) in patients with histologically confirmed stage IV pancreatic cancer receiving first-line systemic chemotherapy, and to compare its prognostic relevance with other psoas-related parameters, including psoas muscle index (PMI) and psoas muscle size. We hypothesized that higher baseline FRPM, reflecting greater ectopic fat deposition within the psoas muscle, would be associated with shorter OS and PFS and would retain prognostic value independent of established clinical and inflammatory prognostic factors.

## 2. Materials and Methods

### 2.1. Study Design and Patient Population

This retrospective, two-center cohort study included patients with histologically confirmed stage IV pancreatic cancer who received first-line systemic chemotherapy between 2016 and 2025 at Ümraniye Training and Research Hospital and Göztepe Prof. Dr. Süleyman Yalçın City Hospital. Consecutive eligible patients were identified through institutional databases. Patients were included if they had histologically confirmed stage IV pancreatic cancer, had received first-line systemic treatment for metastatic disease, and had available baseline clinical, laboratory, and radiologic data suitable for body composition analysis. Patients lacking essential baseline imaging or follow-up data were excluded.

**Figure 1:**
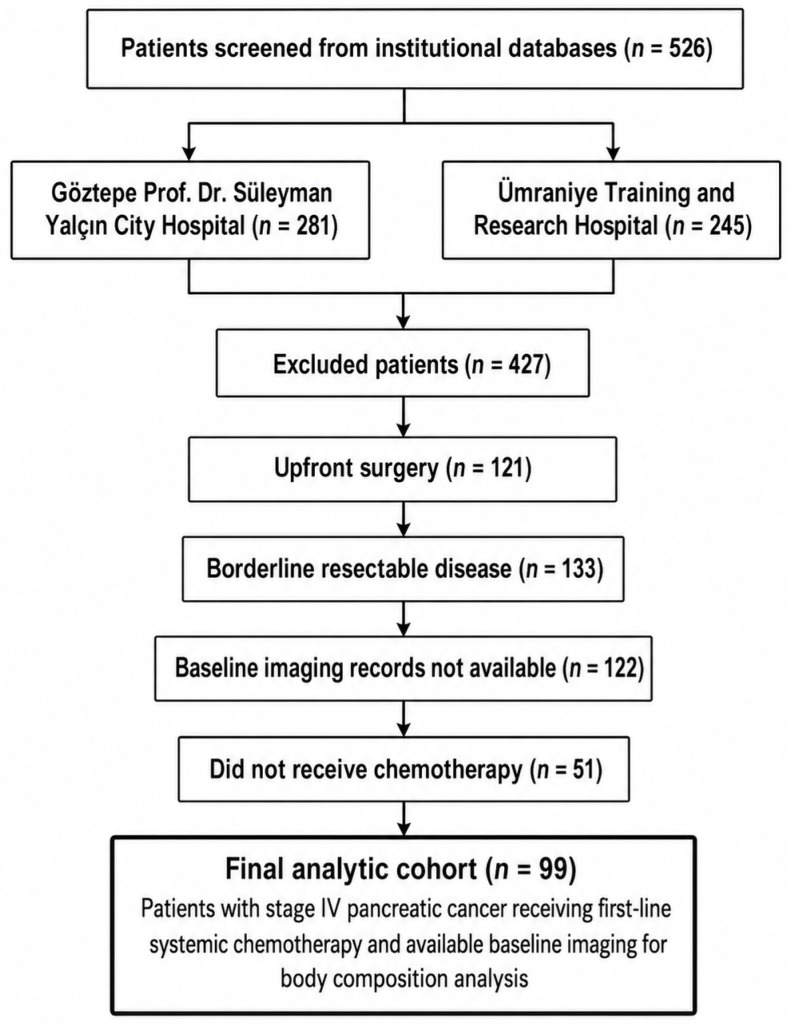
STROBE-compliant patient selection.

A STROBE-compliant patient flow diagram was prepared to summarize the screening process, reasons for exclusion, and final analytic cohort. Overall, 526 patients were screened from institutional databases, including 281 patients from Göztepe Prof. Dr. Süleyman Yalçın City Hospital and 245 patients from Ümraniye Training and Research Hospital. Of these, 427 patients were excluded: 121 because they underwent upfront surgery, 133 because they had borderline resectable disease, 122 because baseline imaging records were not available, and 51 because they did not receive chemotherapy. The final analytic cohort consisted of 99 patients with stage IV pancreatic cancer who received first-line systemic chemotherapy and had suitable baseline CT imaging for body composition analysis (Figure 1).

### 2.2. Data Collection

Demographic, clinical, treatment, and laboratory data were retrieved from medical records. Recorded variables included age, sex, body weight, height, body mass index (BMI), Eastern Cooperative Oncology Group performance status (ECOG PS), smoking and alcohol history, family history, comorbidities, disease presentation at the time of metastatic diagnosis (de novo metastatic vs. recurrent disease), sites and number of metastatic lesions, first-line systemic treatment regimen, best response to first-line therapy, number of treatment lines received, treatment-related adverse events, and survival status.

Baseline laboratory parameters obtained at the time of metastatic disease evaluation included carcinoembryonic antigen (CEA), carbohydrate antigen 19-9 (CA19-9), hemoglobin, neutrophil count, lymphocyte count, platelet count, blood urea nitrogen, creatinine, albumin, total protein, alkaline phosphatase, gamma-glutamyl transferase, aspartate aminotransferase, alanine aminotransferase, total bilirubin, direct bilirubin, lipase, amylase, C-reactive protein (CRP), glucose, calcium, potassium, and sodium.

In addition, inflammation- and nutrition-related indices were calculated, including the neutrophil-to-lymphocyte ratio (NLR), prognostic nutritional index (PNI), systemic immune-inflammation index (SII), and C-reactive protein–albumin–lymphocyte (CALLY) index, where applicable.

### 2.3. Body Composition Assessment

Baseline computed tomography (CT) images obtained before initiation of first-line systemic chemotherapy were reviewed for body composition analysis. The primary body composition parameter of interest was the fat ratio within the psoas muscle (FRPM), which was used as the main study variable representing ectopic fat deposition within the psoas muscle. In addition, psoas muscle size and psoas muscle index (PMI) were recorded as comparative muscle-related parameters.

Computed tomography (CT) images were retrospectively analyzed at the level of the third lumbar vertebra (L3). Bilateral psoas muscles were segmented using 3D Slicer (version 5.10.0) with a semi-automated approach based on Hounsfield Unit (HU) thresholding, followed by manual refinement using brush tools. Psoas muscle area (PSA) was defined using HU thresholds of −29 to 150 HU. Intramuscular adipose tissue was identified using HU thresholds of −190 to −30, and the fat ratio within the psoas muscles (FRPM) was calculated as the proportion of fat area to total muscle area.

For each patient, the left and right psoas muscles were segmented separately and then summed to obtain bilateral psoas measurements. Total psoas cross-sectional area was calculated as the sum of the segmented left and right psoas areas. Intramuscular adipose tissue area was calculated as the sum of fat-density pixels within the bilateral segmented psoas regions. FRPM was calculated using the following equation:

FRPM (%) = [intramuscular adipose tissue area within the bilateral psoas muscles/total bilateral psoas cross-sectional area] × 100.

Psoas muscle index was calculated by normalizing total bilateral psoas muscle area to height squared. FRPM was analyzed primarily as a continuous variable. Sex-specific median-based FRPM groups were used only for descriptive comparisons and Kaplan–Meier visualization and were considered exploratory because these cut-off values were data-derived and not externally validated.

All measurements were performed by a single observer. Patients with poor-quality CT images or conditions potentially affecting accurate muscle assessment, motion artifacts or incomplete visualization of the L3 level were excluded.

### 2.4. Reproducibility Assessment

To assess intraobserver reproducibility of FRPM measurement, a random subset of 29 patients was selected for repeated body composition analysis. The same observer re-measured FRPM in these patients while blinded to the initial measurements and clinical outcomes. One patient was excluded from the reproducibility analysis because of an ambiguous decimal-formatting inconsistency in the repeated measurement record. Therefore, the final reproducibility analysis included 28 patients. Intraobserver reproducibility was evaluated using the intraclass correlation coefficient (ICC) with 95% confidence intervals, based on a two-way mixed-effects, single-measurement, absolute-agreement model. ICC values were interpreted as follows: values below 0.50 indicated poor reliability, 0.50–0.75 indicated moderate reliability, 0.75–0.90 indicated good reliability, and values above 0.90 indicated excellent reliability.

### 2.5. Treatment Response Assessment

Tumor response to first-line systemic treatment was assessed using routine radiologic follow-up performed during clinical practice. Response was categorized as complete response, partial response, stable disease, or progressive disease. When measurable disease and adequate imaging data were available, response assessments were performed according to Response Evaluation Criteria in Solid Tumors version 1.1 (RECIST 1.1).

In routine practice, radiologic assessments were generally performed every 8–12 weeks, or earlier when clinically indicated because of suspected disease progression or clinical deterioration. Progression was determined based on radiologic findings, treating physician documentation, and multidisciplinary clinical evaluation when required. Equivocal cases were reviewed by the investigators using available imaging reports and clinical follow-up records.

### 2.6. Endpoint Definitions

The primary endpoint of the study was overall survival (OS). OS was defined as the time from initiation of first-line systemic chemotherapy to death from any cause or last follow-up.

The secondary endpoint was progression-free survival (PFS). PFS was defined as the time from initiation of first-line systemic chemotherapy to documented disease progression or death from any cause, whichever occurred first. Patients without documented progression or death were censored at the date of last disease assessment. In the present cohort, all patients experienced disease progression during follow-up; therefore, no patients were censored in the PFS analysis.

### 2.7. Statistical Analysis

Statistical analyses were performed using IBM SPSS Statistics, version 26.0 (IBM Corp., Armonk, NY, USA), and R software, version 4.5.3 (R Foundation for Statistical Computing, Vienna, Austria). Continuous variables were summarized as medians and interquartile ranges or means ± standard deviations, according to data distribution. Categorical variables were expressed as numbers and percentages.

FRPM was analyzed primarily as a continuous variable. To improve clinical interpretability, Cox regression results for FRPM were reported per 1-standard deviation increase. For descriptive analyses and Kaplan–Meier visualization, patients were categorized into low- and high-FRPM groups using sex-specific median FRPM values. These grouped analyses were considered exploratory because the cut-off values were data-derived and not externally validated.

Baseline characteristics were compared between exploratory FRPM groups using the Mann–Whitney U test for continuous variables and the chi-square test or Fisher’s exact test for categorical variables, as appropriate. Correlations between FRPM and other continuous variables, including age, BMI, PMI, psoas muscle size, albumin, CRP, NLR, PNI, SII, and CALLY index, were evaluated using Spearman’s rank correlation analysis.

Survival analyses were performed using the Kaplan–Meier method, and survival curves were compared using the log-rank test. Number-at-risk tables and event/censoring counts were added to Kaplan–Meier figures. Cox proportional hazards regression analysis was used to evaluate factors associated with OS and PFS. Hazard ratios and 95% confidence intervals were reported.

Variables with clinical relevance and/or a *p*-value < 0.10 in univariable analysis were considered for multivariable analyses. To avoid overfitting, the main multivariable OS model included FRPM as the primary variable of interest together with age, sex, ECOG performance status, liver metastasis, and CRP. Additional sensitivity analyses were performed to evaluate the robustness of the association between FRPM and OS after adjustment for first-line treatment-regimen category, treatment intensity, treatment center, and calendar period of treatment initiation.

The proportional hazards assumption was assessed using Schoenfeld residuals. The functional form of FRPM was examined using quartile-based analyses and, when feasible, restricted cubic spline models. Missing data were handled using available-case analyses. No imputation was performed because of the retrospective design and modest sample size. All statistical tests were two-sided, and a *p*-value < 0.05 was considered statistically significant.

### 2.8. Ethical Considerations

The study protocol was reviewed and approved by the Umraniye Training and Research Hospital Scientific Research Ethics Committee (Decision No.: 137, approval date: 25 March 2026). The study was conducted in accordance with the principles of the Declaration of Helsinki. Given the retrospective design and the use of existing clinical and imaging data, the requirement for informed consent was waived by the ethics committee.

### 2.9. AI Disclosure Statement

During manuscript revision, ChatGPT (OpenAI, San Francisco, CA, USA, GPT-5.5) was used to assist with language editing and refinement of wording for clarity and readability. The tool was not used to generate original data, perform primary statistical analyses, make clinical interpretations independently, or determine the scientific conclusions of the study. All text edited by AI was critically reviewed, edited, and approved by the authors, who take full responsibility for the accuracy, integrity, and final content of the manuscript.

## 3. Results

Baseline characteristics of the study population according to exploratory sex-specific median-based FRPM groups are summarized in Table 1. Among the 99 included patients, 48 were categorized as having low FRPM and 51 as having high FRPM. Patients in the high-FRPM group were significantly older than those in the low-FRPM group (67.0 vs. 60.5 years, *p* < 0.001). Apart from age, most baseline demographic, disease-related, laboratory, and treatment characteristics were comparable between the two groups. When ECOG performance status was dichotomized as 0–1 versus 2, a numerical imbalance was observed, with ECOG PS 2 being more frequent in the high-FRPM group, although this difference did not reach statistical significance (27.5% vs. 12.5%, *p* = 0.064).

First-line systemic treatment regimens were heterogeneous, reflecting real-world treatment patterns over the long inclusion period. Overall, the most frequently used regimens were FOLFIRINOX (33.3%), gemcitabine plus nab-paclitaxel (18.2%), single-agent gemcitabine (18.2%), and cisplatin plus gemcitabine (14.1%). The distribution of first-line treatment regimens did not differ significantly between the low- and high-FRPM groups (*p* = 0.417); however, given the clinical relevance of treatment heterogeneity, this factor was further evaluated in sensitivity analyses.

Correlation analysis demonstrated that higher FRPM values were associated with older age, higher BMI, and lower psoas muscle quantity-related parameters. Specifically, FRPM showed a moderate positive correlation with age (rho = 0.428, *p* < 0.001) and a weak positive correlation with BMI (rho = 0.299, *p* = 0.003). In contrast, FRPM was inversely correlated with psoas muscle size (rho = −0.431, *p* < 0.001) and showed a weak inverse correlation with PMI (rho = −0.199, *p* = 0.048). No significant associations were observed between FRPM and the evaluated inflammation- or nutrition-related indices, including albumin, CRP, NLR, PNI, SII, and CALLY (Table 2).

Intraobserver reproducibility of FRPM measurement was assessed in 28 patients after exclusion of one case because of an ambiguous decimal-formatting inconsistency in the repeated measurement record. The mean FRPM value was 1.26 ± 0.64 in the initial measurement and 1.18 ± 0.62 in the repeated measurement. The ICC for repeated FRPM measurements was 0.899 (95% CI, 0.79–0.95; *p* < 0.001), indicating good intraobserver reliability.

**Figure 2:**
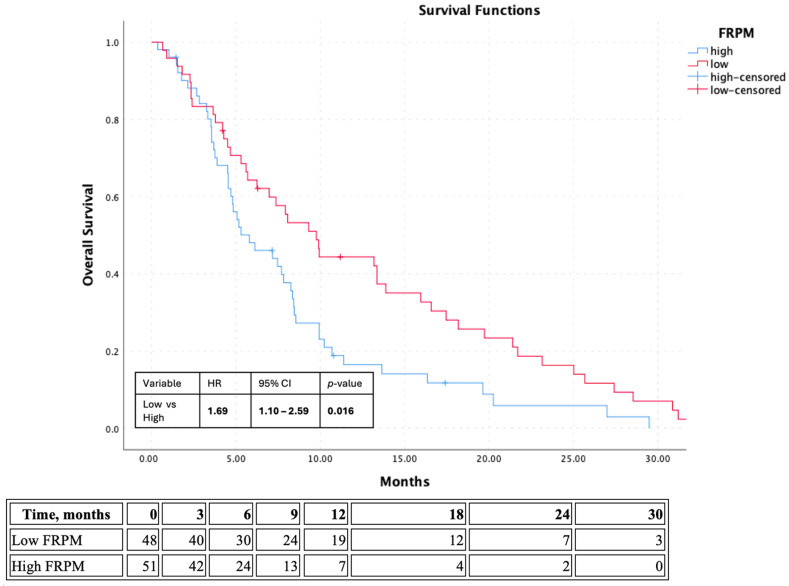
Kaplan–Meier curve for overall survival according to exploratory sex-specific median-based FRPM groups. Number-at-risk table is shown below the curve.

Exploratory Kaplan–Meier analysis according to sex-specific median-based FRPM groups demonstrated a significant difference in OS between the two groups. Median OS was 9.76 months in the low-FRPM group and 5.78 months in the high-FRPM group. Patients with high FRPM had significantly shorter OS than those with low FRPM (log-rank *p* = 0.015) (Figure 2). In grouped Cox analysis, high FRPM was associated with an increased risk of death compared with low FRPM (HR: 1.69, 95% CI: 1.10–2.60, *p* = 0.016). During follow-up, 92 deaths occurred and 7 patients were censored in the OS analysis. Specifically, 45 deaths and 3 censored observations occurred in the low-FRPM group, while 47 deaths and 4 censored observations occurred in the high-FRPM group.

**Figure 3:**
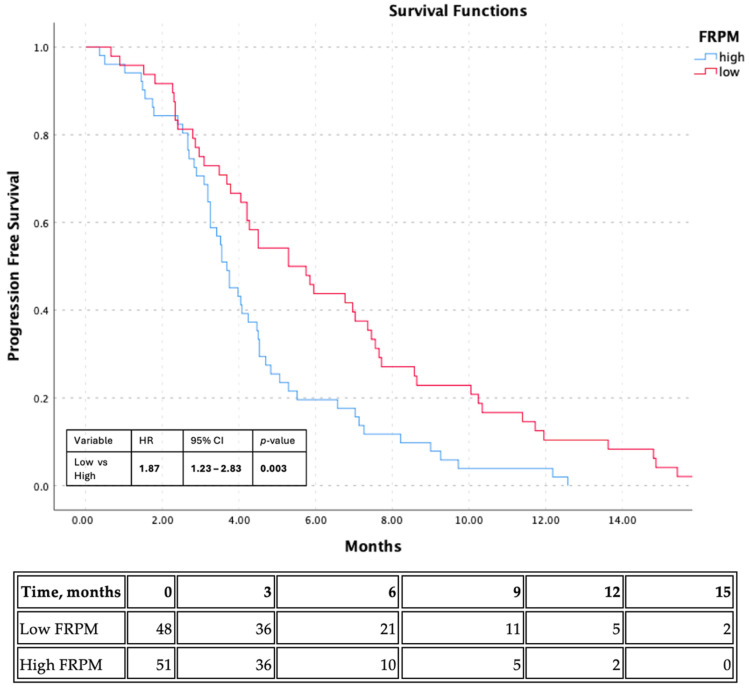
Kaplan–Meier curve for progression-free survival according to exploratory sex-specific median-based FRPM groups. Number-at-risk table is shown below the curve.

Exploratory Kaplan–Meier analysis for PFS also demonstrated a significant difference between the sex-specific median-based FRPM groups. Median PFS was 5.29 months in the low-FRPM group and 3.68 months in the high-FRPM group. Patients with high FRPM had significantly shorter PFS than those with low FRPM (log-rank *p* = 0.003) (Figure 3). In grouped Cox regression analysis, high FRPM was associated with an increased risk of progression or death compared with low FRPM (HR: 1.87, 95% CI: 1.23–2.84, *p* = 0.003). All 99 patients experienced a PFS event during follow-up; therefore, no patients were censored in the PFS analysis. Specifically, 48 PFS events occurred in the low-FRPM group and 51 PFS events occurred in the high-FRPM group.

In univariable Cox regression analysis for overall survival, higher FRPM was significantly associated with worse survival when analyzed as the primary continuous variable and reported per 1-standard deviation increase (HR: 1.27, 95% CI: 1.03–1.56, *p* = 0.028). In exploratory grouped analysis, high FRPM was also associated with an increased risk of death compared with low FRPM (HR: 1.69, 95% CI: 1.10–2.60, *p* = 0.016). In contrast, PMI and psoas muscle size were not significantly associated with overall survival. Among clinical variables, ECOG PS 2 (HR: 2.08, 95% CI: 1.24–3.50, *p* = 0.006), liver metastasis (HR: 2.05, 95% CI: 1.28–3.29, *p* = 0.003), and higher CRP levels (HR: 1.006, 95% CI: 1.001–1.010, *p* = 0.008) were significantly associated with worse overall survival. Albumin and PNI showed borderline associations with survival, whereas age, sex, BMI, treatment center, disease presentation, peritoneal metastasis, metastatic site count, hemoglobin, NLR, SII, and CALLY were not significantly associated with overall survival (Table 3).

**Figure 4:**
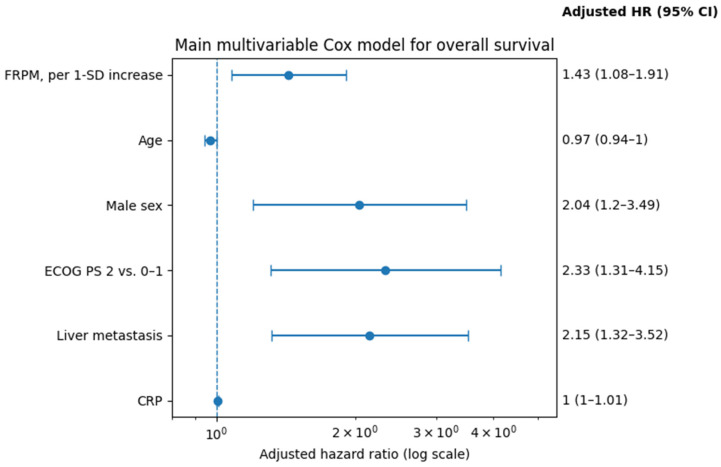
Forest plot of the main multivariable overall survival model.

In the main multivariable Cox regression analysis for overall survival, FRPM remained independently associated with worse survival when analyzed as a continuous variable and reported per 1-standard deviation increase after adjustment for age, sex, ECOG performance status, liver metastasis, and CRP (adjusted HR: 1.43, 95% CI: 1.08–1.91, *p* = 0.014). In the same model, male sex (adjusted HR: 2.04, 95% CI: 1.20–3.49, *p* = 0.009), ECOG PS 2 versus 0–1 (adjusted HR: 2.33, 95% CI: 1.31–4.15, *p* = 0.004), liver metastasis (adjusted HR: 2.15, 95% CI: 1.32–3.52, *p* = 0.002), and higher CRP (adjusted HR: 1.005, 95% CI: 1.000–1.009, *p* = 0.033) were also independently associated with shorter overall survival, whereas age was not statistically significant (adjusted HR: 0.97, 95% CI: 0.94–1.00, *p* = 0.095) (Table 4, Figure 4).

Sensitivity analyses were performed to evaluate the robustness of the association between FRPM and OS after accounting for treatment-related, institutional, and temporal factors. After additional adjustment for first-line treatment-regimen category and treatment intensity, the association between FRPM and OS remained directionally consistent but was attenuated and did not retain conventional statistical significance. In contrast, the association remained statistically significant after additional adjustment for treatment center, calendar period of treatment initiation, and both variables together (Table 5). Because the largest individual regimen subgroup was FOLFIRINOX and only included 33 patients, regimen-specific subgroup Cox analyses were considered underpowered and potentially unstable. Therefore, the effect of treatment heterogeneity was evaluated using treatment-adjusted sensitivity models rather than separate regimen-specific survival models.

The proportional hazards assumption was assessed for the main multivariable OS model using Schoenfeld residuals. No violation of the proportional hazards assumption was observed for FRPM (*p* = 0.225). To further explore the functional form of the association between FRPM and OS, FRPM was also evaluated using quartile-based categories. Compared with the lowest FRPM quartile, the adjusted HRs for OS were 1.47 for quartile 2 (95% CI: 0.80–2.70, *p* = 0.220), 2.10 for quartile 3 (95% CI: 1.02–4.33, *p* = 0.044), and 2.65 for quartile 4 (95% CI: 1.21–5.79, *p* = 0.015), suggesting a stepwise increase in mortality risk across higher FRPM quartiles.

## 4. Discussion

In this retrospective two-center cohort of 99 patients with stage IV pancreatic cancer treated with first-line systemic chemotherapy, we found that higher FRPM was associated with significantly shorter overall survival and progression-free survival. Importantly, FRPM remained independently associated with worse overall survival after adjustment for age, sex, ECOG performance status, liver metastasis, and CRP, whereas PMI and psoas muscle size were not significantly associated with overall survival in univariable analysis. Taken together, these findings suggest that ectopic fat deposition within the psoas muscle may provide prognostic information beyond simple psoas muscle quantity-related measures in metastatic pancreatic cancer. Importantly, the association between FRPM and OS should be interpreted in light of the sensitivity analyses. Although FRPM remained significantly associated with OS in the main multivariable model, the association was attenuated after additional adjustment for first-line treatment-regimen category and treatment intensity and did not retain conventional statistical significance. This finding suggests that part of the observed prognostic association may be influenced by treatment selection and regimen intensity, which are closely related to patient fitness, performance status, comorbidity burden, and real-world clinical decision-making. Nevertheless, the direction of effect remained consistent across treatment-adjusted models, supporting the potential prognostic relevance of FRPM while emphasizing the need for cautious interpretation and external validation.

Our results are broadly consistent with the growing pancreatic cancer literature showing that muscle quality-related parameters may be as important as, or potentially more informative than, measures of muscle quantity alone. The 2024 meta-analysis by Zhang and colleagues concluded that myosteatosis was associated with worse survival outcomes in pancreatic cancer [7], while the 2025 meta-analysis of CT-defined sarcopenia highlighted substantial heterogeneity related to definitions and study populations [5]. Together, these studies suggest that body composition is prognostically relevant in pancreatic cancer, but also that the choice of metric matters. In this context, our findings are notable because FRPM was prognostic, whereas PMI and psoas muscle size were not, supporting the possibility that fat infiltration within muscle may better reflect biologically meaningful frailty than area-based measurements alone.

The present study also aligns conceptually with prior work in advanced gastrointestinal cancers. Ikegami et al. reported that ectopic fat deposition within the psoas muscle had prognostic significance in stage IV gastric cancer patients receiving systemic chemotherapy, which is particularly relevant because their clinical setting closely resembles ours [14]. Although gastric and pancreatic cancers differ biologically, both are cachexia-prone gastrointestinal malignancies, and both may be strongly influenced by host nutritional and metabolic reserves [15]. Our study extends that concept to metastatic pancreatic cancer and suggests that the adverse prognostic effect of psoas fat infiltration may not be limited to gastric cancer.

Several biological mechanisms may explain why higher FRPM was associated with inferior outcomes. Intramuscular fat accumulation is generally considered a marker of reduced muscle quality and impaired functional reserve, and may reflect systemic catabolism, chronic inactivity, metabolic dysregulation, and cancer-related cachexia [16,17]. In pancreatic cancer, where rapid nutritional deterioration and treatment-related vulnerability are common, such a marker may identify patients with reduced capacity to tolerate disease burden or systemic therapy. This interpretation is supported by our correlation analysis in which higher FRPM was associated with older age, higher BMI, and lower psoas quantity-related parameters, suggesting that FRPM captures a phenotype characterized less by simple weight loss alone and more by qualitative deterioration of muscle tissue. In pancreatic cancer, several disease-specific factors may further modify the relationship between FRPM and clinical outcomes. Exocrine pancreatic insufficiency, biliary obstruction, cholestasis, impaired oral intake, cancer-related pain, and early nutritional deterioration may contribute to muscle wasting and intramuscular fat accumulation. In addition, treatment-regimen intensity may influence both survival and the ability of patients to tolerate systemic therapy. These pancreas-specific factors were not systematically captured in the present retrospective dataset, and future studies should evaluate whether they mediate or modify the association between FRPM and survival.

An interesting aspect of our findings is that FRPM was not significantly associated with albumin, CRP, NLR, PNI, SII, or CALLY in correlation analysis, even though CRP was independently associated with overall survival in the multivariable model. This may indicate that FRPM is not merely a surrogate for systemic inflammation or nutritional depletion, but rather reflects a partially distinct dimension of host condition. In other words, FRPM may provide complementary prognostic information alongside more traditional inflammatory or nutritional markers. That possibility strengthens the rationale for integrating body composition metrics into broader prognostic models in metastatic pancreatic cancer. However, this finding should be interpreted cautiously because the modest sample size may have limited the statistical power to detect correlations between FRPM and inflammatory or nutritional markers.

Another clinically relevant observation was that FRPM outperformed PMI and psoas muscle size in survival analyses. This finding is important because psoas-based metrics are attractive in routine practice due to their relative simplicity, but not all psoas-derived measurements are equally informative. Prior literature has raised concerns that psoas-only area may not fully represent whole-body skeletal muscle composition [18,19,20]. Our findings suggest that although psoas area-related measures alone may be limited, qualitative psoas muscle assessment, particularly fat infiltration, may still retain prognostic utility. It is also important to emphasize that FRPM should not be interpreted as a definitive marker of sarcopenia or as a replacement for established sarcopenia definitions. Rather, FRPM represents a CT-derived marker of ectopic fat deposition and muscle quality, conceptually related to myosteatosis. Therefore, FRPM may complement, rather than replace, conventional body composition measures such as skeletal muscle index, psoas muscle index, and muscle radiodensity.

This study has several limitations. First, it was retrospective and involved a relatively modest sample size from two centers, which increases the possibility of selection bias and limits subgroup analyses. Although sensitivity analyses were performed to account for treatment center and calendar period of treatment initiation, residual confounding related to institutional practice patterns, temporal changes in systemic treatment, supportive care, imaging schedules, and clinical documentation cannot be excluded. Second, first-line treatment regimens were heterogeneous across the long inclusion period. Although the association between FRPM and OS remained directionally consistent after adjustment for treatment-regimen category and treatment intensity, the effect was attenuated and did not retain conventional statistical significance, indicating that treatment-related factors may partly influence the observed association. Third, missing data were handled using available-case analyses, and no imputation was performed; therefore, missingness may have introduced additional selection bias. Fourth, although intraobserver reproducibility was good, body composition measurements were performed by a single observer and interobserver variability was not assessed. Fifth, VAT and SAT were not systematically measured at the L3 level; therefore, the incremental prognostic value of FRPM beyond conventional adiposity parameters could not be established. Sixth, the sex-specific median-based FRPM groups were data-derived and used only for exploratory descriptive and Kaplan–Meier analyses; these cut-off values have not been externally validated and should not be interpreted as clinically established thresholds. Finally, external validation in larger, preferably prospective cohorts is required before FRPM can be considered for broader clinical implementation.

Despite these limitations, our study has several strengths. It focused on a clinically well-defined population of stage IV pancreatic cancer patients receiving first-line systemic chemotherapy, used baseline CT imaging obtained in routine care, and evaluated a relatively underexplored muscle-quality biomarker in direct comparison with more conventional psoas-related measures. In addition, FRPM remained prognostic in the main multivariable model and showed directionally consistent associations across sensitivity analyses, suggesting that it may capture clinically relevant host-related information not fully reflected by conventional psoas muscle quantity measures.

In conclusion, higher FRPM was associated with shorter overall and progression-free survival in patients with stage IV pancreatic cancer receiving first-line systemic chemotherapy. FRPM remained significantly associated with OS in the main multivariable model, while treatment-adjusted sensitivity analyses showed directionally consistent but attenuated associations. These findings suggest that ectopic fat deposition within the psoas muscle may represent a promising CT-based marker of adverse prognosis and muscle quality in metastatic pancreatic cancer. Future larger, preferably prospective studies should determine whether FRPM can be standardized, externally validated, compared with conventional adiposity parameters such as VAT and SAT, and integrated with clinical and laboratory markers to improve risk stratification in routine oncology practice.

## Figures and Tables

**Table 1:** Baseline characteristics of the cohort according to exploratory sex-specific median-based FRPM groups.

Variable	Overall (*n* = 99)	Low FRPM (*n* = 48)	High FRPM (*n* = 51)	*p*-Value
Age, years	65.0 (58.0–68.0)	60.5 (54.0–67.0)	67.0 (63.0–69.5)	<0.001
Sex, female	48/99 (48.5%)	23/48 (47.9%)	25/51 (49.0%)	1.000
BMI, kg/m^2^	24.1 (21.4–26.4)	23.7 (21.0–26.1)	24.6 (21.9–27.8)	0.190
De novo metastatic disease	79/99 (79.8%)	36/48 (75.0%)	43/51 (84.3%)	0.249
Liver metastasis	63/99 (63.6%)	27/48 (56.2%)	36/51 (70.6%)	0.138
Peritoneal metastasis	32/99 (32.3%)	15/48 (31.2%)	17/51 (33.3%)	0.825
Lung metastasis	17/99 (17.2%)	10/48 (20.8%)	7/51 (13.7%)	0.349
Non-regional lymph node metastasis	53/99 (53.5%)	28/48 (58.3%)	25/51 (49.0%)	0.353
Ascites	22/99 (22.2%)	9/48 (18.8%)	13/51 (25.5%)	0.420
ECOG PS, 0–1/2	79/99 (79.8%)/20/99 (20.2%)	42/48 (87.5%)/6/48 (12.5%)	37/51 (72.5%)/14/51 (27.5%)	0.064
Number of metastatic sites	2.0 (1.0–2.0)	2.0 (1.0–2.0)	2.0 (1.0–2.0)	0.396
Hemoglobin, g/dL	11.6 (10.3–12.6)	11.6 (10.5–12.5)	11.5 (10.0–12.6)	0.790
Albumin, g/dL	3.6 (3.1–4.2)	3.7 (3.1–4.3)	3.6 (3.2–4.0)	0.212
CRP, mg/L	14.3 (5.5–39.6)	12.5 (4.2–37.0)	17.0 (6.2–54.5)	0.144
CA19-9, U/mL	488.5 (44.1–3634.5)	468.5 (40.5–1897.5)	524.0 (54.4–4773.8)	0.408
NLR	3.3 (2.4–4.7)	3.1 (2.5–4.4)	3.5 (2.4–5.1)	0.627
PNI	45.2 (39.4–50.4)	46.3 (39.2–52.3)	44.5 (39.8–49.4)	0.281
First-line treatment regimen				0.417
FOLFIRINOX	33/99 (33.3%)	21/48 (43.8%)	12/51 (23.5%)	
Gemcitabine + nab-paclitaxel	18/99 (18.2%)	9/48 (18.8%)	9/51 (17.6%)	
FOLFOX	10/99 (10.1%)	4/48 (8.3%)	6/51 (11.8%)	
Cisplatin + gemcitabine	14/99 (14.1%)	6/48 (12.5%)	8/51 (15.7%)	
Gemcitabine + capecitabine	4/99 (4.0%)	1/48 (2.1%)	3/51 (5.9%)	
Gemcitabine	18/99 (18.2%)	6/48 (12.5%)	12/51 (23.5%)	
Capecitabine	2/99 (2.0%)	1/48 (2.1%)	1/51 (2.0%)	

Continuous variables are presented as median (interquartile range), and categorical variables are presented as number (percentage). Patients were categorized into low- and high-FRPM groups according to sex-specific median FRPM values. Comparisons between groups were performed using the Mann–Whitney U test for continuous variables and the chi-square test or Fisher’s exact test, as appropriate. Abbreviations: FRPM, fat ratio within the psoas muscle; BMI, body mass index; ECOG PS, Eastern Cooperative Oncology Group performance status; CRP, C-reactive protein; NLR, neutrophil-to-lymphocyte ratio; PNI, prognostic nutritional index; CA19-9, carbohydrate antigen 19-9; FOLFIRINOX: 5-fluorouracil, folinic acid, irinotecan, oxaliplatin; FOLFOX: 5-fluorouracil, folinic acid, oxaliplatin. These groups were used for descriptive comparisons only and were not intended to represent externally validated clinical cut-off values.

**Table 2:** Correlation of FRPM with body composition and host-related variables.

Variable	Spearman’s Rho	*p*-Value
Age, years	0.428	<0.001
BMI, kg/m^2^	0.299	0.003
PMI	−0.199	0.048
Psoas muscle size	−0.431	<0.001
Albumin, g/dL	−0.088	0.384
CRP, mg/L	0.154	0.127
NLR	0.083	0.415
PNI	−0.047	0.646
SII	0.146	0.150
CALLY	−0.114	0.263

Correlations were assessed using Spearman’s rank correlation analysis. Correlation coefficients are presented as Spearman’s rho. Positive coefficients indicate that higher FRPM values were associated with higher values of the corresponding variable, whereas negative coefficients indicate an inverse association. Abbreviations: FRPM, fat ratio within the psoas muscle; BMI, body mass index; PMI, psoas muscle index; CRP, C-reactive protein; NLR, neutrophil-to-lymphocyte ratio; PNI, prognostic nutritional index; SII, systemic immune-inflammation index; CALLY, C-reactive protein–albumin–lymphocyte index.

**Table 3:** Univariable Cox regression analysis for overall survival.

Variable	HR	95% CI	*p*-Value
FRPM, per 1-SD increase	1.27	1.03–1.56	0.028
High FRPM vs. low FRPM, exploratory grouped analysis	1.69	1.10–2.60	0.016
PMI	0.96	0.85–1.09	0.506
Psoas muscle size	0.99	0.96–1.03	0.780
Age	1.00	0.97–1.03	0.942
Male sex	1.12	0.73–1.71	0.610
BMI	0.98	0.93–1.03	0.420
ECOG PS 2 vs. 0–1	2.08	1.24–3.50	0.006
Recurrent disease vs. de novo metastatic disease	0.70	0.42–1.16	0.166
Liver metastasis	2.05	1.28–3.29	0.003
Peritoneal metastasis	0.77	0.49–1.20	0.249
Lung metastasis	0.91	0.51–1.60	0.733
Non-regional lymph node metastasis	0.93	0.61–1.41	0.721
Ascites	1.05	0.65–1.68	0.849
Number of metastatic sites	1.23	0.96–1.56	0.102
Hemoglobin	1.05	0.94–1.17	0.405
Albumin	0.75	0.54–1.05	0.094
CRP	1.006	1.001–1.010	0.008
CA19-9	1.00002	1.00000–1.00005	0.063
NLR	1.04	0.98–1.10	0.172
PNI	0.97	0.95–1.00	0.053
SII	1.0000001	1.0000000–1.0000003	0.122
CALLY	1.000005	0.999984–1.000027	0.625

Cox proportional hazards regression analysis was performed for overall survival. Hazard ratios (HRs) and 95% confidence intervals (CIs) are presented. FRPM, as a continuous variable, is reported per 1-standard deviation increase. The high- versus low-FRPM comparison represents an exploratory grouped analysis based on sex-specific median-derived groups. For other continuous variables, HRs represent the effect of a 1-unit increase in the corresponding variable. Abbreviations: FRPM, fat ratio within the psoas muscle; PMI, psoas muscle index; BMI, body mass index; ECOG PS, Eastern Cooperative Oncology Group performance status; CRP, C-reactive protein; CA19-9, carbohydrate antigen 19-9; NLR, neutrophil-to-lymphocyte ratio; PNI, prognostic nutritional index; SII, systemic immune-inflammation index; CALLY, C-reactive protein–albumin–lymphocyte index.

**Table 4:** Main multivariable Cox proportional hazards regression analysis for overall survival.

Variable	Adjusted HR	95% CI	*p*-Value
FRPM, per 1-SD increase	1.43	1.08–1.91	0.014
Age	0.97	0.94–1.00	0.095
Male sex	2.04	1.20–3.49	0.009
ECOG PS 2 vs. 0–1	2.33	1.31–4.15	0.004
Liver metastasis	2.15	1.32–3.52	0.002
CRP	1.005	1.000–1.009	0.033

Multivariable Cox proportional hazards regression analysis was performed for overall survival. Hazard ratios (HRs) and 95% confidence intervals (CIs) are presented. FRPM was analyzed as a continuous variable and is reported per 1-standard deviation increase. For other continuous variables, HRs represent the effect of a 1-unit increase in the corresponding variable. Abbreviations: FRPM, fat ratio within the psoas muscle; ECOG PS, Eastern Cooperative Oncology Group performance status; CRP, C-reactive protein.

**Table 5:** Sensitivity analyses for the association between FRPM and overall survival.

Sensitivity Model	Adjusted HR for FRPM per 1-SD Increase	95% CI	*p*-Value
Main model	1.43	1.08–1.91	0.014
Main model + first-line treatment-regimen category	1.32	0.99–1.77	0.060
Main model + treatment intensity	1.30	0.98–1.73	0.073
Main model + treatment center	1.44	1.09–1.91	0.011
Main model + calendar period of treatment initiation	1.47	1.10–1.97	0.010
Main model + treatment center + calendar period of treatment initiation	1.48	1.11–1.97	0.008

The main model included FRPM, age, sex, ECOG performance status, liver metastasis, and CRP. Sensitivity models were constructed by adding the specified variable to the main model. FRPM was analyzed as a continuous variable and is reported per 1-standard deviation increase. Treatment intensity was categorized according to whether patients received intensive combination chemotherapy or less intensive/single-agent treatment. Calendar period was defined according to the period of first-line treatment initiation. Abbreviations: FRPM, fat ratio within the psoas muscle; HR, hazard ratio; CI, confidence interval; ECOG, Eastern Cooperative Oncology Group; CRP, C-reactive protein.

## Data Availability

The datasets generated and/or analyzed during the current study are not publicly available due to institutional privacy regulations and the retrospective use of patient-level clinical data. De-identified data may be made available from the corresponding author upon reasonable request, subject to approval by the relevant institutional authorities and compliance with applicable ethical and legal requirements.
